# A Content Analysis of Vaping Advertisements on Twitter, November 2014

**DOI:** 10.5888/pcd13.160274

**Published:** 2016-09-29

**Authors:** Shaina J. Sowles, Melissa J. Krauss, Sarah Connolly, Patricia A. Cavazos-Rehg

**Affiliations:** Author Affiliations: Melissa J. Krauss, Sarah Connolly, Patricia A. Cavazos-Rehg, Department of Psychiatry, Washington University School of Medicine, St. Louis, Missouri.

## Abstract

**Introduction:**

Vaping has increased in popularity, and the potential harms and benefits are largely unknown. Vaping-related advertising is expected to grow as the vaping industry grows; people are exposed primarily to vaping advertisements on the Internet, and Twitter is an especially popular social medium among young people. The primary objective of our study was to describe the characteristics of vaping-related advertisements on Twitter.

**Methods:**

We collected data on 403,079 English-language tweets that appeared during November 2014 and contained vaping-related keywords. Using crowdsourcing services, we identified vaping-related advertisements in a random sample of 5,000 tweets. The advertisement tweets were qualitatively coded for popular marketing tactics by our research team. We also inferred the demographic characteristics of followers of 4 Twitter handles that advertised various novel vape products.

**Results:**

The random sample of 5,000 vaping-related tweets included 1,156 (23%) advertisement tweets that were further analyzed. Vape pens were advertised in nearly half of the advertisement tweets (47%), followed by e-juice (21%), which commonly mentioned flavors (42%). Coupons or price discounts were frequently observed (32%); only 3% of tweets mentioned vaping as a way to quit smoking or as an alternative to smoking. One handle had a disproportionately high percentage of racial/ethnic minority followers.

**Conclusion:**

Vaping poses a threat to smoking prevention progress, and it is important for those in tobacco control to understand and counter the tactics used by vaping companies to entice their consumers, especially on social media where young people can easily view the content.

## Introduction

“Vaping” is the process of inhaling the vapor produced by converting liquid or plant-based substances into an aerosol product that contains an active ingredient (eg, nicotine, tetrahydrocannabinol [THC], cannabidiol [CBD]), flavoring chemicals, and solvents (eg, vegetable glycerin, propylene glycol) (1). Vaping has recently increased in popularity (2). First-generation vaping devices (ie, electronic cigarettes, or e-cigarettes) closely resembled tobacco cigarettes; however, newer generations of e-cigarettes (ie, personal vaporizers) are high-tech gadgets in various shapes, sizes, and colors, and many are battery-powered and refillable (3).

The potential harms and benefits of vaping are largely unknown (4). Two randomized control trials demonstrated that the use of nicotine-containing vaping devices significantly decreased cigarette consumption and was associated with higher rates of smoking abstinence (5,6). Many vaping proponents argue that vaping is less harmful than traditional smoking (7). Conversely, concern exists that vaping will reignite the social acceptability of smoking in our culture and reverse progress made in denormalizing this behavior (8). Vaping opponents also point out the harms associated with this behavior, such as exposure to nicotine and carcinogens (including formaldehyde) (9); however, more research is required before conclusions can be drawn about the safety of vaping and e-cigarettes.

As the vaping industry expands, we expect to see an increase in vaping-related advertising. The primary objective of our study was to describe the characteristics of vaping-related advertisements on Twitter, a popular social media site with a largely young adult following; 32% of Twitter users are aged 18 to 29 years (10). A study on the marketing of e-cigarettes on Twitter demonstrated that tweets about e-cigarettes were generally positive and that marketing of these products on Twitter is common (11). We build on that study by expanding our analysis to all vaping-related advertisements and characterizing the types of people who follow novel vape-product marketers on Twitter.

## Methods

Informed by previous literature (11), we compiled an initial list of vaping-related keywords. The popularity of these keywords on Twitter was checked by using Topsy.com, a free online service that provides historical estimates of the volume of tweets containing keywords of interest as well as sample tweets containing the keywords of interest. Through these searches on Topsy.com, we identified additional vaping-related keywords and added them to our list. We used in our analysis all keywords that were estimated to have at least 1,000 tweets in the previous 30 days (Box). We then collected all English-language tweets that contained the selected keywords and appeared from November 1 through November 30, 2014, from Gnip (www.gnip.com), a social media data provider that has access to the full archive of public Twitter data. This collection resulted in 403,079 vaping-related tweets. Of these, a random sample of 5,000 was drawn by using SAS proc surveyselect (SAS Institute, Inc) to be examined via content analysis. A similar sample size was used in our previous content analysis of Twitter data (12).

**Box. Vaping-Related Keywords and Hashtags Used to Collect Tweets**

e cig

ehookah

vaper or #vaper

e cigarette

e-hookah

vapers or #vapers

e cigarettes

electronic cigarette

vaping or #vaping

e cigs

electronic cigarettes

vaporizer or #vaporizer

e pen

e-liquid

#vapecommunity

e-cig

eliquid or #eliquid

#vapefam

ecig or #ecig

e-liquids or #e-liquids

#vapejuice

e-cigarette

g pen

#vapelife

e-cigarettes

hookah pen

#vapelyfe

ecigarettes or #ecigarettes

hookah pens

#vapeon

e-cigs

shisha pen

#vapeporn

ecigs or #ecigs

vape or #vape

In the first phase of analysis, we used the crowdsourcing services of CrowdFlower (www.crowdflower.com) to identify vaping-related tweets that contained advertisements that included direct promotions for or reviews of vaping products or shops or stores that sell vaping-related products. Crowdsourcing is “the practice of obtaining needed services, ideas, or content by soliciting contributions from a large group of people and especially from the online community rather than from traditional employees or suppliers” (www.merriam-webster.com). We used CrowdFlower and similar methods in our previous analysis (12). CrowdFlower workers viewed each of the 5,000 randomly selected tweets to determine whether the tweet was a vaping-related advertisement and whether the tweet included an image of the product or an image of a person using the product. Given our interest in novel vaping behaviors, we also asked CrowdFlower to flag tweets about e-hookahs, hookah pens, or vaping marijuana. Final determinations of CrowdFlower workers for 200 randomly selected tweets were compared with the determinations of a research team member, and reliability was excellent for most codes (Krippendorff α for advertisement, 0.90; for product image, 1.00; for person using product, 1.00; for hookah, 1.00) and moderate for the mention of marijuana (Krippendorff α = 0.52).

After advertisement tweets were identified by CrowdFlower, research team members performed an in-depth analysis. The type of product advertised was identified (eg, vape pen, juice or e-liquid, accessories such as carrying case or pen charms, and vape shops). The source (ie, sender) of the tweet was coded as a vape shop, a vaping-related handle other than a shop (ie, vaping-related term in handle name or Twitter bio), or other non-vaping–related source.

A subsample of 500 advertisement tweets was examined by 2 research team members to inductively identify common themes of marketing tactics. Based on this initial examination, all tweets were then coded for the following themes: 1) the use of coupons, price discounts, free giveaways, or contests, 2) mentions of e-juice flavors, 3) mentions or displays of image(s) of colorful vape pen(s), or 4) mentions of using vape products as a way to quit smoking or as a healthy alternative to smoking. Finally, if a link was provided in the tweet, the type of website linked was identified as eBay, Amazon, a vape shop site, or some other website.

The advertisement tweets were coded by 5 research team members. Each tweet was coded by 2 team members independently. Interrater reliability was assessed across all 5 coders and classifications (median Krippendorff α = 0.72; range, 0.55–0.85). Any discrepancies were discussed and resolved.

To gain insight into the people who follow vape-product marketers on Twitter, on May 13, 2016, we used DemographicsPro, a social media analytics company (www.demographicspro.com), to infer the demographic characteristics of followers of 4 Twitter handles that advertised various novel vape products. We chose to focus on novel products in our analysis because e-cigarettes had already been studied (11). To choose 4 Twitter handles, we searched our analyzed sample of vape advertisements for Twitter handles that had a large number of followers and that marketed specific types of vaping-related products, as determined by their Twitter handle profile. The 4 handles chosen represent 1) a company that markets vaporizers (and used vaping terminology rather than “e-cigarettes” in their profile), 2) a company that markets a novel e-cigar, 3) a company that markets e-liquid only, and 4) a company that markets a vape pen known for vaping marijuana.

DemographicsPro uses propriety algorithms to infer demographic characteristics of social media users according to their social media behavior. We used DemographicsPro to infer the demographic characteristics of Twitter users in a previous study (13). To infer demographic characteristics, DemographicsPro uses multiple data signals, including the nature and strength of the company’s social media networks, the information consumed by the user, and the language used in the user’s posts and biographies. DemographicsPro has iteratively tested its models on large, established samples of Twitter users who have verified demographics. In addition to inferring the demographic characteristics of the followers of the vape-related Twitter handles, DemographicsPro provides Twitter benchmark values for comparison purposes. These benchmarks are determined by analyzing followers from a large number of Twitter accounts and calculating the median average value for each demographic characteristic. We descriptively compared the inferred demographic characteristics of followers of the 4 Twitter handles with the Twitter median average.

Washington University’s Human Research Protection Office granted this study a non–human subjects determination, which released this research from institutional review board oversight.

## Results

Among the 5,000 tweets that were randomly selected from the full sample of 403,079 vaping-related tweets, 1,743 (35%) were classified as vaping-related advertisements. Of these tweets, 282 (16%) were sent from @vapedeals, an online discount site based in the United Kingdom. These tweets were removed from analysis to avoiding skewing the sample by overrepresenting the codes for coupons or vaping-related handles. Additionally, a popular retweet advertising CBD oil appeared 305 times (18%) and was removed. The final analytic sample consisted of 1,156 tweets. Most of these tweets (n = 615, 53%), originated from sources other than a vape shop or vaping-related handle; these sources included noncommercial people sharing coupons or deals online, product reviews, and commercial accounts that were not vaping-related but happened to be advertising a vape product (eg, chargers, cases, other accessories). The remaining tweets in the sample were from vape shops (n = 420, 36%) or vaping-related handles (n = 121, 10%). The median number of followers across the 1,156 advertising tweets was 162 (interquartile range, 23–825), and the sum of the followers across the tweets (representing the potential reach to Twitter users) was 3,462,141.

Nearly one-quarter (n = 271, 23%) of tweets contained an image of the advertised vaping product, and 3% (n = 30) depicted a person using the product to vape. Six percent of tweets (n = 74) referenced vaping marijuana rather than nicotine e-liquid or vaping in general (ie, no substance specified); only 2% (n = 22) referenced vaping hookah or shisha.

Vape pens were advertised in 542 (47%) tweets. Of the 542 tweets advertising vape pens, 175 (32%) contained an image of the pen. Of these 175 tweets, 65 (37%) mentioned or pictured a non–neutral-colored pen (Table 1). E-juice or e-liquid was advertised in 247 tweets (21%); of these 247 tweets, 42% (n = 104) mentioned flavors (eg, strawberry, marshmallow, #flavors). A picture of food (eg, vanilla custard, pie, fruit) appeared in 16% (n = 17) of the 104 tweets that mentioned a flavor. Of all 1,156 tweets, 10% (n = 110) promoted vaping-related accessories and 30% (n = 343) promoted either a vape shop in general or did not specify the product being advertised (the product could not be distinguished).

**Table 1:** Examples of Tactics for Marketing Vaping Products Observed in a Sample (n = 1,156) of Advertisement Tweets, November 2014

Theme	No. (%)	Sample Tweets
Offers coupons, price discounts, free giveaways, or contests	373 (32)	• Show us your “I Voted” sticker today and receive 10% off at any Vapor Spot location. #vote #IVoted #vape • Win a Stylish Shisha Pen in a flavour of your choice!
Uses flavors in products to attract customers	104 (9)	• The fresh sweetness of ripe, yellow bananas offers a creamy and tropical flavor. #ecigs #banana • Nice warm vape of Gingerbread Brûlée from @vaporsoven on this crisp Autumnal morning.....Bootiful! #relaxandvapeon
Mentions or shows images of a non–neutral-colored vape pen^a^	65 (6)	• CE4 E-Cig Vaporizors with charge adaptor $25. All colors available. • NEW GENUINE Atmos BULLET 2 GO bullet to go Vaporizer with WARRANTY ALL COLORS
Suggests that products are a quitting aid or alternative to smoking	40 (3)	• you will shrivel up and die if you #smoke, start vaping today at rainbow vapes • 2014 #Ecig is the newest health device for smokers. Switch to smoke free and save money.

a No images provided in this article because of copyright restrictions on reproduction of Twitter images.

Coupons, price discounts, free giveaways, or contests frequently appeared in tweets (n = 373, 32%). Only 3% (n = 40) of tweets mentioned vaping as a way to quit smoking or as an alternative to smoking. We observed a few marketing tactics, such as cartoons and sexual or “manly” references, which have been historically used in tobacco advertising.

A large majority (n = 966, 84%) of tweets contained links to websites. Of the 966 tweets, nearly one-third (n = 288, 30%) linked directly to a vape shop’s website, and 7% (n = 81) linked to an eBay or Amazon page where the product could be purchased. Links to other websites appeared in 411 (43%) of 966 tweets, and approximately 19% (186 of 966) of these links did not work at the time of our analysis.

The number of followers in November 2014 of 4 popular Twitter handles that marketed vaping-related products ranged from nearly 2,000 to more than 100,000 (Table 2). By May 2016, three handles had increased their audience and 1 handle had not.

**Table 2:** Twitter Activity of 4 Popular Twitter Accounts Hosted by Companies That Market Vaping-Related Products and Selected From Our Analyzed Sample (n = 1,156 ) of Vape Advertisements, November 2014^a^

Twitter Handle	Product Focus	No. of Followers in November 2014	No. of Tweets With Vape Key Words in November 2014^b^	No. of Followers in May 2016	Total No. of Tweets Posted Since Joining Twitter	Date Joined Twitter
Handle 1	Vaporizers	12,085	68	30,800	8,274	Nov 2009
Handle 2	E-cigars	14,493	8	14,000	2,833	Apr 2013
Handle 3	E-liquid	1,810	11	5,167	966	Dec 2013
Handle 4	Marijuana vape pens	14,656	154	18,600	13,200	Sep 2012

Abbreviation: E, electronic.

a To choose 4 Twitter handles, we searched our analyzed sample of vape advertisements for Twitter handles that had a large number of followers and that marketed specific types of vaping-related products, as determined by their Twitter handle profile.

b Among all tweets collected with vaping-related keywords (n = 403,079).

The audiences for vaporizers, e-liquid, and marijuana vape pens were disproportionately male compared with the Twitter median average (Figure). Compared with the Twitter median average, followers of the e-liquid and marijuana vape pen handles were more likely to be aged 20 to 24 and followers of the marijuana vape pen and e-cigar handles were more likely to be aged 25 to 29. Only 2% or less of the followers across accounts were aged 16 or younger, but 16% to 31% of the followers across accounts were aged 17 to 19 years; these percentages were similar to the Twitter median averages. Two Twitter handle audiences were disproportionately white (vaporizer and e-liquid) compared with the Twitter median average. In contrast, the marijuana vape pen audience was disproportionately black or Hispanic.

**Figure:**
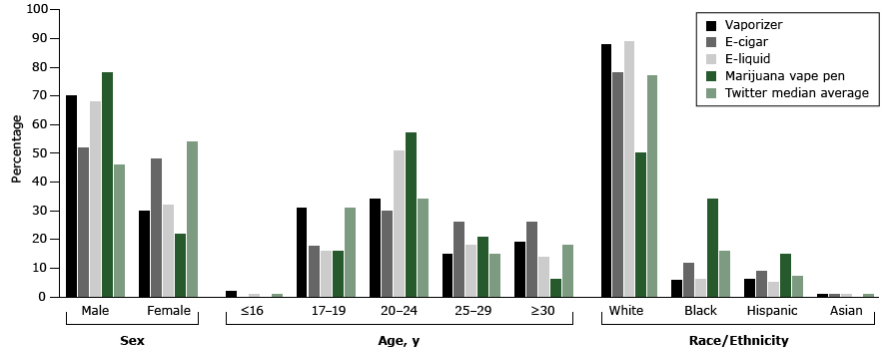
Inferred demographic characteristics of 4 popular Twitter handles that marketed vaping-related products and the Twitter median average (determined by analyzing followers from a large number of Twitter accounts and calculating the median average value for each demographic characteristic) when the analysis was conducted in November 2014. DemographicsPro, a social media analytics company (www.demographicspro.com), was used in May 2016 to determine the inferred characteristics of the followers of the 4 Twitter handles. **Table:** 

**Characteristic**	Vaping-Related Product of 4 Popular Twitter Handles				**Twitter Median Average**
	**Vaporizer**	**E-cigar**	**E-liquid**	**Marijuana Vape Pen**	
**Sex**					
Male	70	52	68	78	46
Female	30	48	32	22	54
**Age**					
≤16 y	2	0	1	0	1
17–19 y	31	18	16	16	31
20–24 y	34	30	51	57	34
25–29 y	15	26	18	21	15
≥30 y	19	26	14	6	18
**Race/ethnicity**					
White	88	78	89	50	77
Black	6	12	6	34	16
Hispanic	6	9	5	15	7
Asian	1	1	1	0	1

## Discussion

This study highlights the presence of vaping-related advertisements on Twitter and identified very few tweets that advertised vaping as a cessation aid. Price discounts or coupons were frequently observed in our sample; this observation is similar to research findings using 2012 Twitter data (11). These incentives can entice potential consumers to make a purchase or help establish a loyal customer base (14). Tobacco control efforts seek to eliminate the use of promotional discounts because smoking behaviors respond to tobacco-price changes (15). A similar behavioral effect is plausible for price changes in e-cigarettes and vaping products (16).

Some cigarette smokers do wish to initiate vaping to help them quit (17); in these instances the use of online promotional incentives to maintain the accessibility and affordability of vaping products could be deemed worthy. However, evidence supporting the safety and efficacy of vaping as an aid to quit smoking conventional cigarettes is lacking (5,6). Smokers who use traditional tobacco products and vaping products are exposed to harms caused by both products (18). E-cigarette use can increase the willingness to smoke traditional cigarettes in a young adult population (19). Thus, the online use of price discounts or coupons that we observed on Twitter is a concerning practice from an industry that is rapidly growing and evolving.

Historically, tobacco advertisements use flavor descriptors and colorful packaging to entice an influx of new consumers, especially young consumers (20). We observed the promotion of flavored e-juices and images of colorful vape pens in our sample of tweets; these promotions and images could grab the attention of potential consumers and entice them to initiate use of these products. The prevalence of vaping is highest among young adults compared with all other adults, and preliminary research suggests that advertising flavored e-cigarettes increases interest in vaping among young people (21). This interest is troubling because the harms of vaping-related products are largely unknown (4) and information about the potential adverse effects of inhaled flavorings is scarce (22). Our observations of colorful vape pens and flavored e-juices in Twitter advertisements are novel observations and advance the understanding of tactics in marketing vaping products. Because roughly one-third of Twitter’s active users are young people aged 16 to 24 (23), it is important for regulatory agencies and public health officials to monitor the types of advertising messages being delivered on this platform.

We also found messages of vaping as a healthier alternative to smoking or as a quitting aid in only 3% of the advertising tweets; another study found that 11% of advertising tweets conveyed these messages (11). This difference is likely attributable to our use of a broader set of vaping-related key words. “Vaping” is a common term among young people, and although many people vape as an alternative to smoking, the low percentage of advertisements that touted vaping products as quitting aids suggests their uptake is not solely driven by a desire among smokers to quit smoking (24). Our demographic analysis of followers of popular vape-product marketers found that followers were typically in their 20s. Vaping uptake may be driven by perceptions of vaping as a hobby or a networking or socializing opportunity (24). Thus, to better target prevention efforts, it may be important for future studies to delineate the extent to which the vaping industry is directing their marketing toward nonsmoking young adults who may have an interest in vaping for enjoyment or as a hobby rather than a smoking cessation tool.

The Twitter account for the marijuana vaping product had a high percentage of black and Hispanic followers. The use of marijuana has increased significantly in these 2 groups during the past decade (25). Vaping marijuana is an increasingly popular alternative to more traditional methods of using marijuana, especially in states with medical marijuana laws (26). People tend to view vaping marijuana as a safer (ie, reduces impact on the respiratory system) and more cost-effective way to use marijuana (27). The perception that vaping marijuana is safe is problematic because it may foster earlier initiation of marijuana use among underage people or more frequent consumption (26); both underage use and more frequent consumption are risk factors for dependence and abuse (28).

This study had several limitations. First, because the study was exploratory, it examined only a small sample of vaping-related tweets during 1 month; a larger sample of tweets over a longer period of time may have altered our findings. However, the use of generic vaping-related key words in addition to the key word “e-cigarettes” allowed us to create a wider snapshot of vaping-related advertising on Twitter. Second, geolocation data were not available for most tweets in our sample; therefore, we could not determine the country of origin of tweets. This information would be useful in understanding how future US vaping-related regulations may or may not have oversight on the types of advertisements visible on a globally used social media site such as Twitter. Third, a comprehensive examination of the content contained in the external links would have added to the overall understanding of how vaping is being advertised online, but that level of analysis was outside the scope of our study. Such an analysis should document whether the externally linked websites use any age restrictions to prevent viewing the website or purchasing products online. However, minors are able to purchase vaping-related products online even when websites have age verification procedures in place (29).

Vaping poses a threat to the great strides made in curbing the initiation of conventional cigarette use among young people. It is therefore important for those in tobacco control to become knowledgeable about the advertising practices of vaping-product companies, especially on social media where young people can easily view content. In 2016, the Food and Drug Administration finalized a rule to expand its oversight to include the marketing of vaping devices (30). Under this new rule, vaping products will be regulated in the same way as traditional cigarettes, including but not limited to restricting vaping product sales to people aged 18 years or older, using health-warning labels on packages and advertisements, and banning the distribution of free samples. Although this rule is a success for tobacco control, additional legislation is needed to expand the Prevent All Cigarette Trafficking Act, which prohibits the online sale of cigarettes to minors (31), to include vaping devices. Many tweets in our sample linked directly to websites that offered the advertised product for purchase, and research shows that minors can easily purchase vaping products online (29). Thus, additional research is needed on how to reduce the exposure of young people to vaping advertising and how to best verify that underage people cannot purchase such products online. This research will be essential in circumventing a new generation of nicotine-addicted people.
